# Investigating the effects of additional truncating variants in DNA-repair genes on breast cancer risk in *BRCA1*-positive women

**DOI:** 10.1186/s12885-019-5946-0

**Published:** 2019-08-08

**Authors:** Ilnaz Sepahi, Ulrike Faust, Marc Sturm, Kristin Bosse, Martin Kehrer, Tilman Heinrich, Kathrin Grundman-Hauser, Peter Bauer, Stephan Ossowski, Hana Susak, Raymonda Varon, Evelin Schröck, Dieter Niederacher, Bernd Auber, Christian Sutter, Norbert Arnold, Eric Hahnen, Bernd Dworniczak, Shan Wang-Gorke, Andrea Gehrig, Bernhard H. F. Weber, Christoph Engel, Johannes R. Lemke, Andreas Hartkopf, Huu Phuc Nguyen, Olaf Riess, Christopher Schroeder

**Affiliations:** 1 Institute of Medical Genetics and Applied Genomics, University of Tübingen, Tübingen, Germany; 2 CENTOGENE AG, Rostock, Germany; 3 Centre for Genomic Regulation (CRG), The Barcelona Institute of Science and Technology, Barcelona, Spain; 4 Universitat Pompeu Fabra (UPF), Barcelona, Spain; 5 Institute of Medical and Human Genetics, Charité Universitätsmedizin Berlin, Berlin, Germany; 6 Institute for Clinical Genetics, Dresden, Germany; 7 Department of Obstetrics and Gynaecology, Düsseldorf University Hospital, Düsseldorf, Germany; 8 Department of Human Genetics, Hannover Medical School, Hannover, Germany; 9 Institute of Human Genetics, University Hospital Heidelberg, Heidelberg, Germany; 10 Department of Gynaecology and Obstetrics and Institute of Clinical Molecular Biology, University Hospital of Schleswig-Holstein, Christian-Albrechts-University of Kiel, Kiel, Germany; 11 Centre for Hereditary Breast and Ovarian Cancer, University of Cologne and University Hospital Cologne, Cologne, Germany; 12 Institute of Human Genetics, University Hospital Münster, Münster, Germany; 13 Department of Gynaecology and Obstetrics, University Hospital Ulm, Ulm, Germany; 14 Centre of Familial Breast and Ovarian Cancer, Department of Medical Genetics, Institute of Human Genetics, University Würzburg, Würzburg, Germany; 15 Institute of Human Genetics, University of Regensburg, Regensburg, Germany; 16 Institute for Medical Informatics, Statistics and Epidemiology, University of Leipzig, Leipzig, Germany; 17 Institute of Human Genetics, University of Leipzig Hospitals and Clinics, Leipzig, Germany; 18 Department of Obstetrics and Gynecology, University of Tuebingen, Tuebingen, Germany; 19 Department of Human Genetics, Ruhr-University Bochum, Bochum, Germany

**Keywords:** Breast cancer, Age at onset, DNA-repair genes, Next-generation-sequencing, Panel sequencing, Extreme phenotypes, Hereditary breast and ovarian cancer, *BRCA1*, DNA-repair

## Abstract

**Background:**

Inherited pathogenic variants in *BRCA1* and *BRCA2* are the most common causes of hereditary breast and ovarian cancer (HBOC). The risk of developing breast cancer by age 80 in women carrying a *BRCA1* pathogenic variant is 72%. The lifetime risk varies between families and even within affected individuals of the same family. The cause of this variability is largely unknown, but it is hypothesized that additional genetic factors contribute to differences in age at onset (AAO). Here we investigated whether truncating and rare missense variants in genes of different DNA-repair pathways contribute to this phenomenon.

**Methods:**

We used extreme phenotype sampling to recruit 133 *BRCA1*-positive patients with either early breast cancer onset, below 35 (early AAO cohort) or cancer-free by age 60 (controls). Next Generation Sequencing (NGS) was used to screen for variants in 311 genes involved in different DNA-repair pathways.

**Results:**

Patients with an early AAO (73 women) had developed breast cancer at a median age of 27 years (interquartile range (IQR); 25.00–27.00 years). A total of 3703 variants were detected in all patients and 43 of those (1.2%) were truncating variants. The truncating variants were found in 26 women of the early AAO group (35.6%; 95%-CI 24.7 - 47.7%) compared to 16 women of controls (26.7%; 95%-CI 16.1 to 39.7%). When adjusted for environmental factors and family history, the odds ratio indicated an increased breast cancer risk for those carrying an additional truncating DNA-repair variant to *BRCA1* mutation (OR: 3.1; 95%-CI 0.92 to 11.5; *p*-value = 0.07), although it did not reach the conventionally acceptable significance level of 0.05.

**Conclusions:**

To our knowledge this is the first time that the combined effect of truncating variants in DNA-repair genes on AAO in patients with hereditary breast cancer is investigated. Our results indicate that co-occurring truncating variants might be associated with an earlier onset of breast cancer in *BRCA1*-positive patients. Larger cohorts are needed to confirm these results.

**Electronic supplementary material:**

The online version of this article (10.1186/s12885-019-5946-0) contains supplementary material, which is available to authorized users.

## Background

Breast cancer is the most common cancer among women with 30% of all new cancer diagnoses [1]. About one out of eight US women will develop breast cancer during her lifetime. It is estimated that hereditary genetic factors explain 5–10% of all breast cancer cases [2]. In the mid-1990s, *BRCA1* and *BRCA2* [3–5] which are part of the DNA-repair machinery [6] were identified to play a crucial role in hereditary breast and ovarian cancer (HBOC) [3–5, 7, 8]. Together, pathogenic variants in these two genes explain about 24% (95%-CI,23.4 to 24.6%) of all HBOC cases [7]. *BRCA1* and *BRCA2* are functioning as genome guardians by playing a central role in the homologous recombination repair (HRR) pathway. Up to now, more than 300 gene products have been associated with the DNA-repair machinery and genome integrity maintenance of which 25 genes [8] have been linked to HBOC.

In female *BRCA1* mutation carriers, the risk of developing breast cancer by the age of 80 is 72% [9]. Moreover, the incidence of breast cancer rises quickly in early adulthood until age 30 to 40 years in *BRCA1* mutation carriers [9]. Even though pathogenic variants in *BRCA1* are associated with the highest penetrance of HBOC, the cause for the inter-individual and even intra-familial variation in penetrance is not clear and remains an active field of research. This variation results in difficulties in risk calculation and genetic counseling. Several environmental factors such as birth cohort [10], age at menarche [11], number of pregnancies [12], therapeutic abortion [13], oral contraceptives [14], and prophylactic oophorectomy [15, 16] are suspected to affect the risk of cancer in *BRCA1/2* mutation carriers. Using data from the Generations Study, Brewer and colleagues showed that having a first-degree female relative with breast cancer increases the relative risk of breast cancer as compared to those without family history [17]. Moreover, the variation in penetrance can be due to allelic variation, which means variation in the variant type (truncating or missense) and position within the coding region of the *BRCA1* gene [18]. As proposed by Thompson and Easton in 2001 and 2002 and also Rebbeck et al. [19–21], the position of the respective causative pathogenic variant within the coding region of *BRCA1/2* can change breast or ovarian cancer risk. In this context, Rebbeck and colleagues identified three putative “breast cancer cluster regions” including BCCR1 which overlaps with the RING domain of the *BRCA1* protein and an “ovarian cancer cluster region” located in exon 11 [21]. Furthermore, pathogenic variants towards the 3′-end of *BRCA1* lead to a lower risk of ovarian cancer compared to breast cancer [22].

Another cause of differences in penetrance are modifying genes [18]. The Consortium of Investigators of Modifiers of *BRCA1/2* (CIMBA, http://ccge.medschl.cam.ac.uk/consortia/cimba) screened more than 20,000 mutation carriers and performed Genome Wide Association Studies (GWAS) to identify genetic modifier loci [23–29] and described several candidates; each adding a small part of risk variation in *BRCA1* mutation carriers (in total 2.2% in *BRCA1*) [23]. The CIMBA consortium suggested using a combination of different modifier loci to increase the precision of risk prediction. Unlike GWAS studies that are based on common variants, this study pursued the goal to predict *BRCA1* penetrance and AAO of breast cancer by analysing rare variants in genes that are part of the DNA damage response and genome integrity maintenance pathways as well as genes which are interacting with *BRCA1.* Accurate prediction of AAO can become of clinical relevance in order to prevent overtreatment of carriers who will never develop breast cancer during their lifetime or may develop it later in life. To address this issue, we aimed to investigate the differences in AAO of breast cancer among *BRCA1* mutation carriers by studying 311 DNA-repair genes which are contributing to genome stability along with *BRCA1* and *BRCA2*.

## Methods

### Selection of samples for extreme phenotype sampling

Out of more than 30,000 HBOC index cases registered in the German Consortium for Hereditary Breast and/or Ovarian Cancer (GC-HBOC) biobank, 133 *BRCA1*-positive patients either with a personal history of breast cancer below the age of 35 years (early AAO onset) or without personal history of breast cancer at the age of 60 years (controls) were selected for this study. Patients who had undergone prophylactic mastectomy or prophylactic oophorectomy before the age of 45 years were excluded from the analysis [30]. Participants had signed a written informed consent and the study was approved by the local ethics committee (ethic vote number 053/2017BO2). Relevant information regarding age at menarche, number of pregnancies, and Oral contraceptive use was collected from the GC-HBOC database.

### Sequencing and data analysis

Reviewing published literature, genes were considered on the basis of a reported breast cancer association. In addition, all DNA-repair pathway genes were selected from KEGG GENES database (http://www.genome.jp/kegg/genes.html, last accessed: 26.11.2013; Additional file 1: Table S1). A target region of 895.2 kbp consisting of 311 genes was sequenced in total. The coding regions and exon-intron boundaries ±25 bps were targeted (using default parameters of Agilent SureDesign, except for Masking = Most Stringent) and enriched using Agilent SureSelect custom RNA probes (Agilent, Santa Clara, CA). Two hundred nanograms of genomic DNA were checked for quality and quantity by Qubit dsDNA Assay (Thermo Fischer Scientific, Waltham, MA, USA) and fragmented using a Covaris system (Covaris, Inc., Woburn, Massachusetts) to generate fragments of 120–150 base pairs length. Quality and fragment size of sheared DNA were checked using a TapeStation (Agilent, Santa Clara, CA). Sequencing libraries were constructed according to the Agilent SureSelectXT protocol. The pre-capture and post-capture libraries were quantified by a TapeStation. Libraries were sequenced either on a Miseq (Illumina, San Diego CA), NextSeq500 (Illumina, San Diego CA) or HiSeq2500 (Illumina, San Diego CA) platform using paired-end reads of 151 bps or 101 bps.

MegSAP, a free-to-use open-source bioinformatics pipeline was used for data analysis (version 0.1–379-gb459ce0, https://github.com/imgag/megSAP). In brief, adapter and quality trimming was applied using SeqPurge [31]; sequencing reads were mapped to the human genome version GRCh37 with BWA (v. 0.7.15) [32], and ABRA2 [33] (v. 2.05) was used for indel realignment; variant calling was performed by freebayes (v. 1.1.0) [34] and variant annotation was done using snpEff/SnpSift (v. 4.3i) [35]. Quality control was executed on three layers of information including raw reads, mapped reads and variants (Additional file 2: Table S2). We used Alamut batch (v. 1.5.1, Interactive Biosoftware) for splice site annotation.

### Variant interpretation

Variants were automatically classified according to an algorithm based on a modified version of the American College of Medical Genetics and Genomics (ACMG) guidelines for variant classification [36]. According to this algorithm, splice variants at the position +/− 1 and +/− 2 are classified as likely pathogenic if the variant disrupts the function of the gene product unless the population frequency of the variant is not compatible for a pathogenic variant (minor allele frequency of 1% was used as a cutoff). For intronic variants located outside of the canonical splice sites including Cartegni splice sites [37] we referred to Alamut Visual (Interactive Biosoftware) incorporated prediction tools such as MaxEntScan, Splice Site Finder Like, and Human Splicing Finder. Variants were considered as pathogenic or likely pathogenic (collectively termed as pathogenic) if they led to a truncation, initiation loss or canonical splice site effect or if there was a relevant publication in favor of pathogenicity and if there was additional evidence in public database like ClinVar. In case there was no evidence such as functional assessment data available, missense, synonymous and intronic variants were classified as variants of unknown significance (VUS), benign or likely benign according to the Minor Allele Frequency (MAF > 1%) in the 1000 Genomes Project (1KGP), dbSNP, Exome Aggregation Consortium (ExAC) or ESP6500.

### Statistical analysis

Descriptive statistics such as medians, means and standard deviations for continuous data and proportion and 95%-CI for categorical data was used to characterize the study population and sequencing results. A multivariable logistic regression was carried out to control for the potential confounding effect of family history, age at menarche, parity, and use of oral contraceptives. Missing data was imputed using median or mode. The variable *additional truncating DNA-repair variants* was coded as *yes* if the patient carried a truncating DNA-repair variant and it was coded as *no* if the patient was not carrying a truncating DNA-repair variant. The outcome was considered the incidence of cancer. The regression analysis was performed in R 3.5.2. Using GraphPad Prism version 6.07 for Windows (GraphPad Software, La Jolla California USA), we performed Fisher’s exact test to compare the mutational location in each cohort. All *p*-values were two-tailed and *p*-values less than 0.05 were considered to be statistically significant. Maftools was applied to visualize *BRCA1* pathogenic variants with a modified database [38].

### Rare variant association study

Variants obtained from freebayes in VCF format (see above) were annotated using the eDiVA platform (https://ediva.crg.eu/) in order to obtain functional annotation (exonic, nonsynonymous, synonymous, splicing etc.), European population allele frequencies from 1KGP, Exome Variant Server (EVS) and ExAC databases, as well as functional impact scores from CADD. Variants not annotated as ‘exonic’ or ‘splicing’, as well as variants within segmental duplication (SegDup identity > = 0.9) were removed from further analysis. We performed sample quality control by screening for outliers in (a) number of variants per sample and (b) transition to transversion ratio per sample. Second, we calculated the first 10 PCA components of all samples using only synonymous SNVs that were not in linkage disequilibrium and had an allele frequency above 0.005 in EVS. Finally, we compared the rare variant load per gene between the early AAO cohort and controls. No outliers were detected in any QC test and early AAO patients and controls were clustering in a single group in the PCA. Following QC, we removed any variant with European AF higher than 0.01 in any of the three databases: EVS, 1KGP, and ExAC. Additionally, we excluded all variants annotated as synonymous or with a CADD score below 10 (considered neutral). Using the remaining rare, likely damaging variants we performed Burden and SKAT-O association tests implemented in the R package SKAT (https://www.hsph.harvard.edu/skat/download/) version 1.3.0. The Null model for both tests was computed using the SKAT_Null_Model function with output set to dichotomous outcome (out_type = “D”) and no sample adjustment (Adjustment = FALSE). For the SKAT-O test we used the SKATBinary function with default parameters except for method that was set to “optimal.adj” (equivalent to SKAT-O method). Minor allele frequencies (MAF) of variants transformed with Get_Logistic_Weights were used as weights. The burden test was performed using the same function (SKATBinary) and parameters, except for method that was set to “Burden”.

## Results

### Participants characteristics

In total, 133 *BRCA1* positive women were screened for truncating variants in 311 DNA-repair genes. The cohort with early AAO consisted of 73 women who developed breast cancer at an age younger than 35 years (median age at onset, 27 years; interquartile range (IQR) 25–27 years). The controls consisted of 60 participants, cancer-free by the age of 60 years. Follow-up data showed that some developed breast cancer at an age older than 60 years (*n* = 25; 41.7%) with a median age at onset of 64 years (IQR, 62–67) or had no history of breast cancer (*n* = 35; 58.3% median age, 70 years; IQR, 63–75 years). The demographic characteristics of the participants are shown in Table 1.

**Table 1:** Demographic characteristics of the population study

	Early age at onset cohort	Control cohort
Total Number	73	60
Breast cancer positive	100%	41.7%
Median age at onset(IQR)	27 (25–27)	64 (62–67) *n* = 25
*BRCA1* variant location		
BCCR1 (95%-CI)	13.8% (6.1–25.4%)	11.5% (4.4–23.4%)
BCCR2 (95%-CI)	8.6% (2.9–19.0%)	5.8% (1.2–15.9%)
BCCR2’ (95%-CI)	22.4% (12.5–35.3%)	15.4% (6.9–28.1%)
OCCR (95%-CI)	25.9% (15.3–39%)	42.3% (28.7–56.8%)
*BRCA1* variant type % (95%-CI)		
Frame-Shift-Del	26.0% (16.5–37.6%)	35.0% (23.1–48.4%)
Frame-Shift-Ins	19.2% (10.9–30.1%)	16.7% (8.3–28.5%)
Missense variant	8.2% (3.1–17.0%)	13.3% (5.9–24.6%)
Nonsense variant	26.0% (16.5–37.6%)	21.7% (12.1–34.20%)
Splice-Site variant	5.5% (1.5–13.4%)	5.0% (1.0–13.9%)
CNV	15.1% (7.8–25.4%)	8.3% (2.8–18.4%)
Family History *Data available for*	73 (100%)	60 (100%)
First-degree relative with Breast and/or Ovarian cancer	41 (56.2%)	59 (98.4%)

*BCCR* Breast cancer cluster region, *BCCR1* c.179–505, *BCCR2* c.4328–4945, *BCCR2’* c.5261–5563, *OCCR* c.1380–4062, *Del* Deletion, *Ins* Insertion, *CNV* Copy number variation

**Fig. 1:**
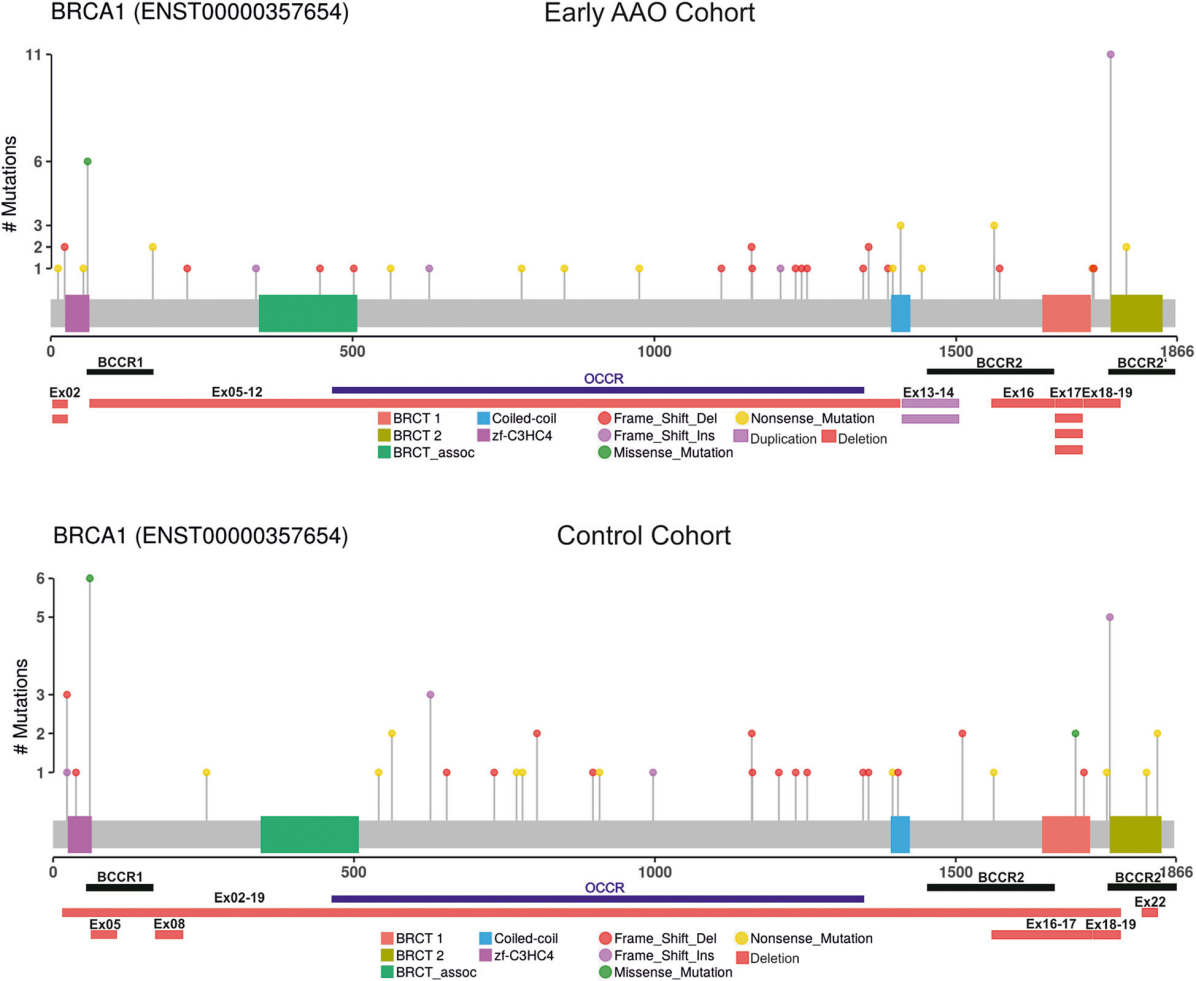
BRCA1 pathogenic variants**.** X axis shows the amino acid position and functional domains of the BRCA1 protein. Each lollipop represents a pathogenic variant and the type of variant is depicted with different colors. The Y axis demonstrates the number of mutation carriers. The Horizontal bars show the copy number variation. Deletion (red) and duplication (purple) is depicted by different colors. Breast cancer Cluster Regions (BCCRs) are shown as black bars and Ovarian Cancer Cluster Region (OCCR, Rebbeck and colleagues [21]) are depicted in dark blue. Splice-site variants are not shown

In total, 117 patients from both cohorts carried a *BRCA1* pathogenic single nucleotide variant (SNV), 13 patients carried a large deletion, and three patients carried a large duplication in *BRCA1* (Fig. 1). In the early AAO cohort, 15.1% of all participants carried a frameshift founder mutation [39] in exon 20 of the *BRCA1* gene (ENST00000357654: c.5266dupC:p.Gln1756fs). The European founder missense variant [40] in exon 4 (ENST00000357654: c.181 T > G: p.Cys61Gly) was the most frequent (10%) pathogenic variant found in the control cohort (Additional file 3: Table S3). All pathogenic variants in *BRCA1* were confirmed by NGS.

With respect to family history, the majority of patients in the control cohort had at least one first-degree relative with breast and/or ovarian cancer as compared to the early AAO patients (56.2% versus 98.4%). Women with larger families who reached older ages are expected to have more relatives with breast and/or ovarian cancer on average in comparison to those whose families are smaller and younger. This can explain the difference between family history of early AAO cohort and control cohort (Table 1).

### Comparison of type and location of *BRCA1* pathogenic variants

To compare allelic variation in type and location of pathogenic variants across the BRCA1 protein between the early age at onset and the control cohort, we compared the pathogenic variant accumulation in different regions of *BRCA1*. Whereas no differences were detected for the Breast Cancer Cluster Regions (BCCRs), which are associated with increased risk of breast cancer (Additional file 4: Figure S1a), differences were found for the Ovarian Cancer Cluster Region (OCCR). 22 (45.3%) patients in the control cohort (Fig. 1, Table 1) carried a pathogenic variant within the OCCR compared to 15 (25.9%) of patients in the early AAO cohort, though the statistical significance was not reached (*p*-value = 0.07). Patients with large deletions or insertions and splice site variants were excluded from this analysis since they either span more than one region or their impact on protein function is not certain, respectively. In the early AAO cohort, 56 patients (76.7%; 95%-CI 65.4 to 85.3%) of *BRCA1* mutation carriers carried a truncating variant while 6 patients (8.2%; 95%-CI 3.1 to 13.3%) carried a missense pathogenic variant (ENST00000357654: c.181 T > G: p.Cys61Gly) and 11 patients (15.1%; 95%-CI 7.8 -25.4%) carried a copy number variation (CNV). In contrast, 47 patients (78.3%; 95%-CI.65.8% to 87.9) carried a truncating variant in controls, 8 patients (13.3%; 95%-CI 5.9 to 24.6%) carried a missense pathogenic variant (Additional file 4: Figure S1b) including ENST00000357654: c.181 T > G: p.Cys61Gly, and c.5096G > A: p.Arg1699Gln and 5 patients (8.3%; 95% CI 2.8 to 18.4%) carried a CNV.

### Truncating germline variants in DNA-repair genes

We evaluated 311 genes that maintain genome integrity and/or have been associated with HBOC. The mean sequencing depth was 456x ± 197.3 SD. Additional file 2: Table S2 shows the detailed results and quality parameters of sequencing. A total of 3703 variants was identified and of those 43 (1.2%) truncating variants (Additional file 5: Table S4) were detected in 36 DNA-repair genes. The affected genes were mainly Single Strand Break Repair genes (SSBR, 30.6%), Double Strand Break Repair genes (DSBR, 30.6%), and check-point factor genes (11.1%). The remaining truncating variants were identified in genes with other functions such as *BRCA1/2* interactors, centrosome formation and signal transduction. In overall, 42 women had at least one additional DNA-repair truncating variant. In the early AAO cohort, 26 out of 73 patients (35.6%; 95%-CI 24.7 - 47.7%) carried at least one additional truncating variant and two cases carried two additional truncating variants in DNA-repair genes (Additional file 6: Figure S2a). Among controls, 16 out of 60 participants carried an additional DNA-repair germline truncating variant (26.7%; 95%-CI 16.1 to 39.7%). In this cohort, three participants carried two germline DNA-repair truncating variants; at least one of them affected a DSBR pathway gene (Additional file 6: Figure S2b).

We investigated the effect of additional DNA-repair truncating variants on the risk of developing breast cancer among *BRCA1* mutation carriers, adjusted for age at menarche, oral contraceptive use, parity and family history. Despite the fact that it did not reach the conventionally accepted *p*-value of 0.05, the odds ratio is in favor of increased breast cancer risk for double heterozygote patients (OR: 3.1; 95% CI 0.92 to 11.5, *p*-value = 0.07). To confirm the validity of our model, the same analysis was carried out on a subset of subjects who were matched for family history (early AAO cohort; *n* = 41 and control cohort; *n* = 59) adjusted for age at menarche, oral contraceptive use and parity (OR: 3.3; 95%-CI 0.92 to 13.3; *p*-value = 0.07). Consistent results were obtained for this subset of cohorts.

**Fig. 2:**
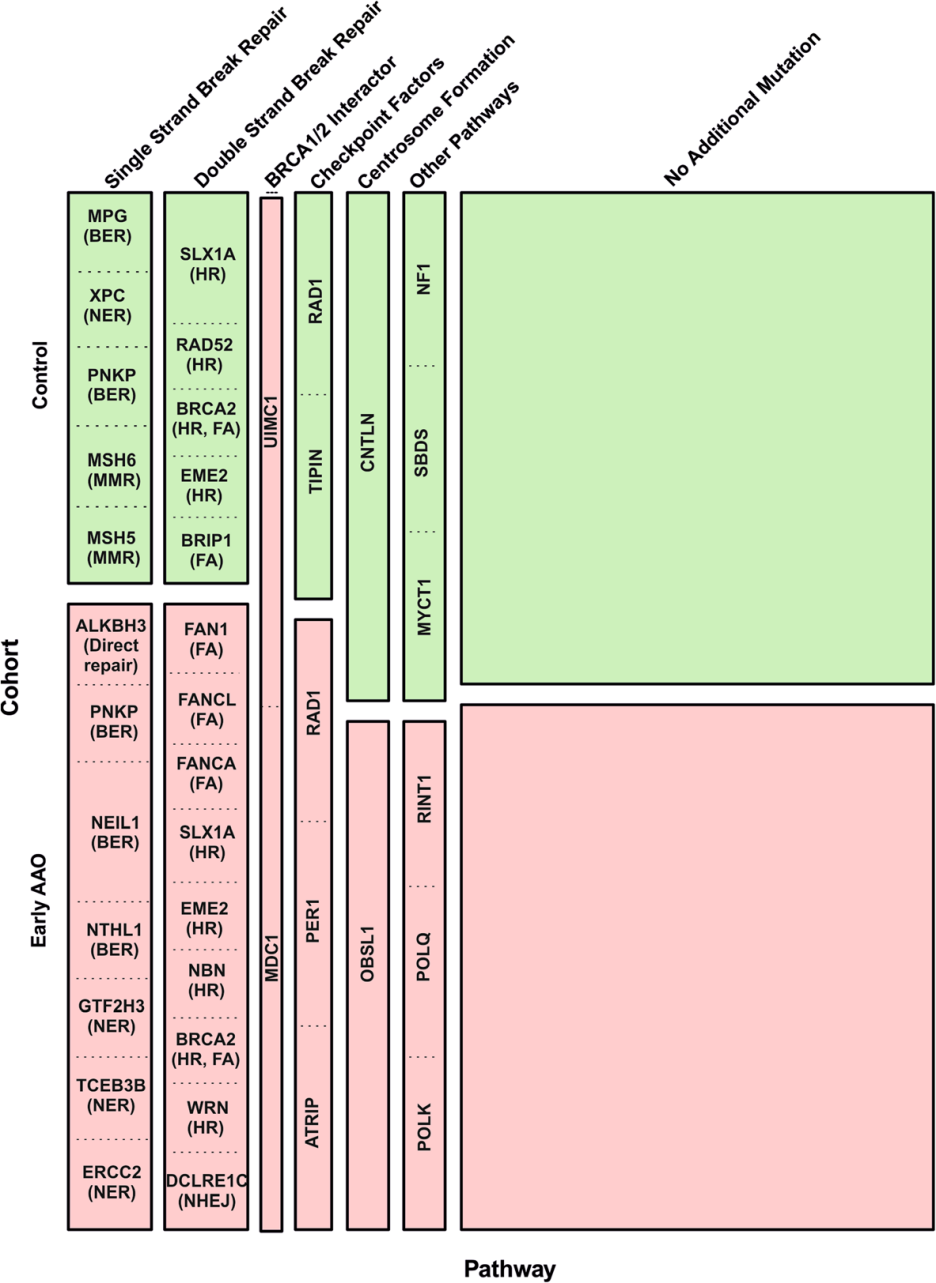
Distribution of carriers of additional DNA-repair mutation in each cohort regarding the type of pathway. 43 truncating variants were detected in 36 DNA-repair genes. These truncating variants mainly affected double-strand break repair (DSBR), single-strand break repair (SSBR), *BRCA1/2* interactors, centrosome formation, and check-point factors. No significant difference was found in DSBR, SSBR, *BRCA1* / *BRCA2* interactors, checkpoint factors and other pathways mutational load between the two cohorts. Two cases in the early AAO cohort carried an additional mutation in *BRCA1* / *BRCA2* interactor genes while no mutation acrrier in these genes was found in control cohort. The width of each block referes to the porportion of mutated pathway among all mutated pathways and the hight of each block referes to the porportion of mutated samples in each cohort. Mutated genes in each pathways are shown in boxes

To test the effect of additional truncating variants in specific DNA-repair pathways, we compared the mutational load in DSBR and SSBR genes between the two cohorts. Among the early AAO cohort, 8/73 women (11.0%; 95%-CI 4.9 -20.5%) carried an additional truncating variant in DSBR compared to 5/60 women (8.3%; 95%-CI 2.8 -18.4%) in the control cohort. Regarding the SSBR genes, we found 8/73 women (11.0% %; 95%-CI 4.9 -20.5%) in the early AAO cohort carrying additional SSBR truncating variants as compared to 5/60 women (8.3%; 95%-CI 2. %-20.5) in the control cohort. The mutational load in DSBR and SSBR did not differ between both cohorts (Fig. 2). Further comparison has been carried out between SSBR- and DSBR- mutation carriers with non-carriers (Additional file 7: Figure S3; Additional file 8: Table S5). In none of the cases differences were statistically significant.

### Pathological characteristics

Among control cohort, 25 (41.7%) patients developed breast cancer at a median age of 64. For these patients the tumor characteristics were compared with the tumor characteristics of the early AAO patients. The immunohistochemical staining of estrogen and progesterone receptors did not differ significantly with respect to the AAO, though the ER and PR negativity was more frequently found in the early AAO cohort compared to affected control patients (*p*-value = 0.28 and 0.76 respectively, Table 2). Tumors of the early AAO group tended to show a higher histological grade compared to the tumors of the affected control patients (Table 2) although the difference failed to reach the significant level (*p*-value = 0.24). Expression of estrogen and progesterone receptors, grading of tumors and histological types of tumors were not significantly different between patients with additional truncating variants in DNA-repair genes and patients without additional DNA-repair truncating variants (Additional file 9: Table S6).

**Table 2:** Histopathological characteristics of tumors

	Early AAO cohort Number (%)	Affected controls Number (%)	*P* value
Histological Type *Data available*	*62 out of 73*	*22 out of 25*	
Ductal	53 (85 .5%)	22 (100%)	0.10
Medullary	6 (9.7%)	0	
Lobular	2 (3.2%)	0	
Others	1 (1.6%)	0	
Histological grade *Data available*	*66 out of 73*	*22 out 25*	
Grade III	53 (80.3%)	14 (63.7%)	0.24
Grade II	13 (19.7%)	7 (31.8%)	
Grade I	0	1 (4.5%)	
Steroid receptors *Data available*	*64 out of 73*	*22 out 25*	
ER negative	47 (73.4%)	13 (59.1%)	0.28
PR negative	52 (81.3%)	17 (77.3%)	0.76
Human Epidermal Receptor *Data available*	*52 out of 73*	*19 out of 25*	
HER2/neu negative	49 (94.2%)	17 (89.5%)	0.60
Triple Negative Breast Cancer *Data Available*	*55 out of 73*	*20 out of 25*	
TNB	32 (58.2%)	11 (55%)	

Data were available for 67 out of 73 patients in the early age at onset cohort and from 25 cases that developed breast cancer in control cohort

*ER* Estrogen receptor, *PR* Progesterone receptor, *HER2* Human epidermal growth factor receptor 2

### Rare variant association study (RVAS)

To assess the load of rare missense (VUS + pathogenic variants) variants in DNA-repair genes on the AAO of breast cancer in *BRCA1*-positive patients we performed a Burden test and a SNP-set (sequence) Kernel Association Test (SKAT-O). To this end, a comprehensive quality control of early AAO cohort and controls was done (see Methods). No differences were observed between early AAO cohort and controls in (a) variants per sample, (b) rare variant load per gene, (c) transition-transversion ratio, and (d) top 10 PCA components. Next, we removed all common variants (MAF > 1% in EVS, 1KGP, or ExAc) as well as all synonymous variants from both early AAO and control cohort. To search for genes conveying an increased risk, we used patients of the early AAO cohort as cases and patients of the late AAO cohort as controls (Additional file 10: Table S7). Although there was no significant gene identified after FDR correction, several genes showed significant un-corrected *p*-values in at least one of the two RVAS tests, requiring more investigation in independent larger cohorts. These candidate genes include *MYBBP1A* (early AAO: 13, controls: 3), *MRE11* (7:0), *TDG* (5:0), *WRN* (7:1), *TP53BP1* (10:3) and *REV1* (8:2) as well as one potential risk reducing factor, *PTCH1* (early AAO: 1, controls: 8).

### Patients with both heterozygous pathogenic variants in *BRCA1* and *BRCA2*

Interestingly, two cases carrying pathogenic variants in both *BRCA* genes were found in either cohort. Case 1 was a patient affected with breast cancer at the age of 26 yrs. She had two first-degree relatives with breast cancer. There was no ovarian cancer and no second-degree relative with any type of cancer. She carried a *BRCA1* pathogenic variant (ENST00000357654: c.1016dupA) and an additional *BRCA2* pathogenic variant (ENST00000544455.1: c.3585_3686delAAAT). Unfortunately, tumor characteristics were not available for this patient. Case 2 was diagnosed with breast cancer at the age of 63.9 years. Her family history was indicative for HBOC: A first-degree relative with breast cancer and three first-degree relatives with ovarian cancer. Also, there was a second-degree relative with breast cancer. She carried a nonsense variant in *BRCA1* (ENST00000357654: c.1687C > T) and a nonsense variant in *BRCA2* (ENST00000544455.1: c.8875G > T). An additional truncating variant was found in *EME2*, (ENST00000568449: c.541_544delGCTG) a DSBR gene. The immunohistochemical staining showed a triple negative tumor.

## Discussion

Genome-wide case control association studies identified susceptibility variants and modifiers of penetrance for *BRCA1* mutation carriers [23, 25–29]. Despite the fact that each modifier explains a small proportion of genetic variation of breast cancer development in carriers of *BRCA1* pathogenic variants [23], still a large proportion of risk variation is unknown. The effect of each modifying variant can be combined into poly genic risk scores (PRSs), which may confer larger relative risks [25, 41]. The approach taken in this study was to enrich for rare variants via preferentially selecting the carriers who are most informative cases [42]. For this reason, the extreme ends of age at onset of hereditary breast cancer were chosen and we aimed to identify differences in the mutational load in these two highly selected cohorts. We hypothesized that inherited truncating variants in DNA-repair genes, which are partner components of *BRCA1* in the maintenance of genome integrity, are likely to interact with *BRCA1* by reducing the age at onset of hereditary breast carcinoma.

Previously reported by Thompson and Easton in 2001 and subject of a more recent study by Rebbeck et al. (2015), it was found that allelic variation in *BRCA1* pathogenic variants is one of the reasons of variation in risk for breast cancer compared to ovarian cancer in HBOC patients. Rebbeck and colleagues described multiple regions associated with a higher risk for breast cancer compared to ovarian cancer (breast cancer cluster regions = BCCRs) and, one region with an increased risk for ovarian cancer compared to breast cancer (OCCR) [19–21]. The mutational position comparison in our cohorts showed no difference for BCCRs but a non-significant higher variant load in the OCCR (*p*-value = 0.07) among controls. Although the difference was not statistically significant, it is worth considering that pathogenic variants in OCCR not only lead to increased risk of ovarian cancer but they also decrease the risk of breast cancer [21]. Regarding the variant type, there was no difference in truncating or missense variants distribution in each cohort. While the most common pathogenic missense variant in both cohort was ENST00000357654: c.181 T > G: p. Cys61Gly, the missense variant ENST00000357654: c.5090G > A: p.Arg1699Gln was exclusively found in two of the patients in the control cohort. This is in line with previous reports where this variant had reduced cumulative risk of breast cancer by age 70 to 20% [43, 44].

Concerning the sum effect of truncating DNA-repair variants on the risk of breast cancer among *BRCA1* mutation carriers, our results are suggesting an increase in the breast cancer risk for the *BRCA1* mutation carriers who carry additional truncating DNA-repair variants (OR: 3.1; 95% CI 0.92 to 11.5; *p*-value = 0.07). The small number of old cancer-free *BRCA1* mutation carriers was a limiting factor in this study. The sum effect of pathogenic variants in DNA-repair genes can lead to a different cancer phenotype as shown by Pritchard and colleagues [45] who reported a higher prevalence of germline DNA-repair pathogenic variants in metastatic prostate cancer patients compared to localized prostate cancer. More recently, Brohl and colleagues [46] reported a significantly higher frequency of germline DNA-repair pathogenic variants in patients with Ewing sarcoma in comparison with general population. By pathway analysis they uncovered that hereditary breast cancer genes, and remarkably, genes involved in DSBR were highly mutated.

Despite the small sample size, we carried out a rare variant association study (RVAS) using SKAT-O and Burden tests to shed light on the role of rare variants in the genetic risk of hereditary breast cancer. The results of SKAT-O and Burden tests were not statistically significant after multiple testing corrections. The top ranked gene in the Burden test is *MRE11*. Mre11 is a member of MRN (MRE11, RAD50, and NBS1) complex [47]. This complex is involved in the sensing of DNA double strand breaks and it initiates the processing of double strand break repair [48–50]. Studies showed that hypomorphic mutations in *MRE11* and *NBS1* lead to *Ataxia telangiectasia* disorder and Nijmegen breakage syndrome, a rare autosomal recessive disorder [51, 52]. Pathogenic variants in the MRN complex were also linked to cancer predisposition. Recently Gupta and colleagues showed an association between triple negative breast cancer and MRE11 defects [53]. The top ranked gene in SKAT-O test and the third top ranked gene in burden test is *MYBBP1* which inhibits colony formation and tumorigenesis and enhances the anoikis in a p53 dependent manner [54].

We also evaluated the tumor histology and immunohistochemical characteristics of the tumors and whether they were influenced by AAO among *BRCA1* mutation carriers. Although the clinicopathological features of *BRCA1* associated breast tumors are studied widely and previous studies showed that *BRCA1* positive tumors demonstrated higher tumor grade, lower estrogen receptor expression, and lower progesterone receptor expression [55–57], the status of ER and PR expression among young and older *BRCA1* associated breast cancer patients is less well studied. Vaziri and colleagues [58] observed that the ER and PR negativity was more common in *BRCA1*-positive patients with an age at onset younger than 50 years compared to above 50 years of age. In 2005, Eerola and colleagues [59] showed similar results by studying *BRCA1/2* positive families in comparison with *BRCA1/2* negative families. They observed a significant difference in ER negativity for *BRCA1* positive, premenopausal patients (age of diagnosis below 50 years). These patients also suffered from higher-grade tumors compared to postmenopausal patients. Our results also demonstrate that carrying a truncating variant in DNA-repair genes in addition to a *BRCA1* pathogenic variant does not change tumor characteristics since the differences in histology and histochemical features of tumors did not differ in those with additional truncating variants in DNA-repair genes compared to those without.

As part of the study we also identified double heterozygotes for pathogenic *BRCA1* and *BRCA2* variants. While the frequency of pathogenic variants in *BRCA1* and *BRCA2* is high in the Ashkenazi Jewish population [60, 61], it was found that 0.3% of all Ashkenazi Jewish breast cancer patients were double heterozygotes for *BRCA1/2* pathogenic variants [62]. In contrast, double heterozygosity for the two major breast cancer genes is expected to be less common phenomenon in other populations. Several studies reported double heterozygous females including a report by Heidemann and colleagues (2012), showing that double heterozygotes were not younger at the time of first diagnosis compared to other patients. Interestingly, they reported a more severe phenotype in double heterozygote females in comparison with their single heterozygote relatives [63]. In the present study, we identified two cases with double heterozygosity in *BRCA1/2*. One of them was found in early AAO cohort whereas another double heterozygote *BRCA1/2* female had a late breast cancer manifestation. These results advocate panel testing, since panel testing allows detection of variants in different genes simultaneously. The presence of additional truncating variants is also of high relevance for the families and segregation analysis should be offered in families with known pathogenic variants to identify patients with high risk for cancer predisposing syndromes.

## Conclusions

In the last few years, several attempts were made to elucidate the variable penetrance of *BRCA1* pathogenic variants. GWA analyses identified several loci, which can modify the penetrance of *BRCA1/2* pathogenic variants and the age at onset of hereditary breast and ovarian cancer to some extent. To our knowledge, this is the first time that germline truncating variants in DNA-repair pathways were studied for their effect on age of breast cancer onset among *BRCA1* carriers. The odds ratio observed in this study indicates a potential effect of co-occurring DNA-repair truncating variants and pathogenic variants in *BRCA1* on the earlier onset of breast cancer. Limitations of this study are the small sample size due to low numbers of asymptomatic *BRCA1* mutation carriers and the large number of missense variants in DNA-repair genes which are of uncertain significance. Further studies and larger cohorts are needed to confirm the results obtained in this study.

## Additional files

Additional file 1 : **Table S1** List of 311 DNA repair and cancer predisposition syndrome genes as well as the pathways. DSBR: Double Strand Break Repair, SSBR: Single Strand Break Repair, HR: Homologous Recombination, NER: Nucleotide Excision Repair, BER, Base Excision Repair, FA: Fancony Anemia, NHEJ: Non-Homologous End Joining. (XLSX 20 kb)

Additional file 2 : **Table S2** The quality parameters of Next Generation Sequencing. (DOCX 14 kb)

Additional file 3 : **Table S3** BRCA1 pathogenic variants. (DOCX 22 kb)

Additional file 4 : **Figure S1** Comparison of type and location of BRCA1 pathogenic variants in two cohorts: a) Accumulation of pathogenic variants in BCCR (Breast Cancer Cluster Region) and OCCR (Ovarian Cancer Cluster Region) are compared in both cohorts. b) Comparison of type of pathogenic variants in two cohorts; Del: deletion; Ins: insertion; CNV: Copy Number Variation. (TIFF 13270 kb)

Additional file 5 : **Table S4** List of putative truncating variants in DNA-repair genes. 43 truncating variants were detected in 36 DNA-repair genes. (XLSX 12 kb)

Additional file 6 : **Figure S2** Additional truncating variants carriers vs non-carriers . The lollipop plot shows the position of BRCA1 pathogenic variants in two cohorts: (a) early AAO and (b) Control cohort; with and without additional truncating variant in DNA-repair genes. X axis shows the functional domain of BRCA1 protein and amino acid position and Y axis demonstrates the number of carriers. Each lollipop represents the location of a BRCA1 pathogenic variant of those with (red) and without (blue) additional truncating variants . Horizontal bars depict the copy number variations of those with (red) and without (blue) additional truncating variant. Splice-site variants are not shown. (TIFF 13653 kb)

Additional file 7 : **Figure S3** Comparison of AAO between DSBR/SSBR gene mutation carriers and non-carriers. (TIFF 18234 kb)

Additional file 8 : **Table S5** Comparison of AAO between carriers of DSBR and SSBR truncating variants in both cohorts. DSBR: Double Strand Break Repair; SSBR: Single Strand Break Repair. (DOCX 14 kb)

Additional file 9 : **Table S6** Comparison of histopathological characteristics of DNA-repair mutation carriers with non-carriers. There was no significant difference in tumors of patients carrying additional truncating variant in DNA-repair genes compare to non-carriers in each cohort. ER: Estrogen receptor; PR: Progesterone receptor; HER2: Human Epidermal growth factor receptor 2. (DOCX 16 kb)

Additional file 10 : **Table S7** The top 8 genes that stood out in the Burden test. q value after FDR correction. (DOCX 13 kb)

## Data Availability

The dataset produced or analyzed in this study is not publicly available due to privacy reasons but it will be available from the corresponding author upon reasonable request.

## Abbreviations

1KGP: 1000 Genomes Project
AAO: Age at (cancer) onset
BCCR: Breast cancer cluster region
*BRCA1*: Breast Cancer 1 gene
CNV: Copy number variation
CPS: Cancer predisposing syndrome
DSBR: Double Strand Break Repair
ER: Estrogen
HBOC: Hereditary breast and ovarian cancer
HER2: Human epidermal growth factor receptor 2
Indel: Insertion/Deletion
OCCR: Ovarian cancer cluster region
PR: Progesterone
RHR: The Ratio of Hazard Ratio
SNV: Single Nucleotide Variation
SSBR: Single Strand Break Repair
VUS: Variant of Unknown Significance
