# Supplementary material for: Investigating the effects of additional truncating variants in DNA-repair genes on breast cancer risk in BRCA1-positive women
Source: BMC Cancer. 2019 Aug 8;19:787. doi: 10.1186/s12885-019-5946-0 (PMC6686546; doi:10.1186/s12885-019-5946-0)
Supplement: Supplementary file 2 — : Table S2 The quality parameters of Next Generation Sequencing. (DOCX 14 kb) [file 12885_2019_5946_MOESM2_ESM.docx]

**Table S2.** **The quality parameters of Next Generation Sequencing**

| Quality Control Parameter | Average for the Panel |
| --- | --- |
| Read counts | 9.7± 5.6 Mio |
| Depth | 456.0± 197.3 |
| 20x Coverage percentage | 97.8± 0.3% |
| Insert size | 178.4± 8.4 |
| On target reads percentage | 68.26± 4.4 |
| Q30 base percentage | 95.13± 1.7 |
