# Supplementary material for: Investigating the effects of additional truncating variants in DNA-repair genes on breast cancer risk in BRCA1-positive women
Source: BMC Cancer. 2019 Aug 8;19:787. doi: 10.1186/s12885-019-5946-0 (PMC6686546; doi:10.1186/s12885-019-5946-0)
Supplement: Supplementary file 3 — : Table S3 BRCA1 pathogenic variants. (DOCX 22 kb) [file 12885_2019_5946_MOESM3_ESM.docx]

**Table S3**. BRCA1 pathogenic variants

| Genomic position | Start | End | Variant Type | Coding region (ENST00000357654) | Protein Change | Early AAO cohort | Control cohort |
| --- | --- | --- | --- | --- | --- | --- | --- |
| chr17 | 41197784 | 41197784 | Stop -gain | c.5503C>T | Arg1835* | no | yes |
| chr17 | 41199678 | 41199678 | Stop-gain | c.5449G>T | Glu1817* | no | yes |
| chr17 | 41201136 | 41201136 | Splice-donor | c.5406+2delT |  | no | yes |
| chr17 | 41201198 | 41201198 | Stop-gain | c.5346G>A | Trp1782* | yes | no |
| chr17 | 41201199 | 41201199 | Stop-gain | c.5345G>A | Trp1782* | yes | no |
| chr17 | 41209079 | 41209079 | Frameshift | c.5266dupC | Gln1756fs | yes | yes |
| chr17 | 41209095 | 41209095 | Stop-gain | c.5251C>T | Arg1751* | no | yes |
| chr17 | 41215361 | 41215361 | Frameshift | c.5182delA | Met1728fs | yes | no |
| chr17 | 41215363 | 41215366 | Frameshift | c.5177_5180delGAAA | Arg1726fs | yes | no |
| chr17 | 41215364 | 41215364 | Stop-gain | c.5179A>T | Lys1727* | yes | no |
| chr17 | 41215392 | 41215392 | Splice-acceptor | c.5153-2delA |  | yes | no |
| chr17 | 41215890 | 41215890 | Splice-donor | c.5152+1G>C |  | yes | no |
| chr17 | 41215906 | 41215906 | Frameshift | c.5137delG | Val1713fs | no | yes |
| chr17 | 41215947 | 41215947 | Missense | c.5096G>A | Arg1699Gln | no | yes |
| chr17 | 41222939 | 41222939 | Splice region | c.4986+6T>C |  | yes |  |
| chr17 | 41223187 | 41223217 | Frameshift | c.4714_4744delTCTGATGACCCTGAATCTGATCCTTCTGAAG | Ser1572fs | yes | no |
| chr17 | 41223242 | 41223242 | Stop-gain | c.4689C>G | Tyr1563* | yes | yes |
| chr17 | 41226489 | 41226490 | Frameshift | c.4533_4534delCA | His1511fs | no | yes |
| chr17 | 41234451 | 41234451 | Stop-gain | c.4327C>T | Arg1443* | yes | no |
| chr17 | 41234556 | 41234556 | Stop-gain | c.4222C>T | Gln1408* | yes | no |
| chr17 | 41234568 | 41234568 | Frameshift | c.4210delC | Leu1404fs | no | yes |
| chr17 | 41242963 | 41242963 | Stop-gain | c.4183C> | Gln1395* | yes | yes |
| chr17 | 41242984 | 41242988 | Frameshift | c.4158_4162delCTCTC | Ser1387fs | yes | no |
| chr17 | 41243480 | 41243483 | Frameshift | c.4065_4068delTCAA | Asn1355fs | yes | yes |
| chr17 | 41243513 | 41243513 | Frameshift | c.4035delA | Glu1346fs | no | yes |
| chr17 | 41243789 | 41243792 | Frameshift | c.3756_3759delGTCT | Ser1253fs | no | yes |
| chr17 | 41243805 | 41243817 | Frameshift | c.3731_3743delATAGCACCGTTGC | His1244fs | yes | no |
| chr17 | 41243844 | 41243848 | Frameshift | c.3700_3704delGTAAA | Val1234fs | yes | no |
| chr17 | 41243923 | 41243923 | Frameshift | c.3624dupA | Leu1209fs | yes | no |
| chr17 | 41243936 | 41243936 | Frameshift | c.3612delA | Ala1206fs | no | yes |
| chr17 | 41244057 | 41244067 | Frameshift | c.3481_3491delGAAGATACTAG | Glu1161fs | no | yes |
| chr17 | 41244063 | 41244063 | Frameshift | c.3485delA | Asp1162fs | yes | yes |
| chr17 | 41244214 | 41244217 | Frameshift | c.3331_3334delCAAG | Gln1111fs | yes | no |
| chr17 | 41244557 | 41244557 | Frameshift | c.2989_2990dupAA | Asn997fs | no | yes |
| chr17 | 41244625 | 41244625 | Stop-gain | c.2923C>T | Gln975* | yes | no |
| chr17 | 41244826 | 41244826 | Stop-gain | c.2722G>T | Glu908 | no | yes |
| chr17 | 41244862 | 41244863 | Frameshift | c.2685_2686delAA | Pro897fs | no | yes |
| chr17 | 41244997 | 41244997 | Stop-gain | c.2551G>T | Glu851* | yes | no |
| chr17 | 41245136 | 41245137 | Frameshift | c.2411_2412delAG | Gln804fs | no | yes |
| chr17 | 41245210 | 41245210 | Stop-gain | c.2338C>T | Gln780* | yes | yes |
| chr17 | 41245239 | 41245239 | Stop-gain | c.2309C>A | Ser770* | no | yes |
| chr17 | 41245347 | 41245351 | Frameshift | c.2197_2201delGAGAA | Glu733fs | no | yes |
| chr17 | 41245587 | 41245587 | Frameshift | c.1961delA | Lys654fs | no | yes |
| chr17 | 41245670 | 41245670 | Frameshift | c.1874_1877dupTAGT | Val627fs | yes | yes |
| chr17 | 41245861 | 41245861 | Stop-gain | c.1687C>T | Gln563* | yes | yes |
| chr17 | 41245927 | 41245927 | Stop-gain | c.1621C>T | Gln541* | no | yes |
| chr17 | 41246040 | 41246044 | Frameshift | c.1504_1508delTTAAA | Leu502fs | yes | no |
| chr17 | 41246212 | 41246212 | Frameshift | c.1336delA | Arg446fs | yes | no |
| chr17 | 41246531 | 41246531 | Frameshift | c.1016dupA | Val340fs | yes | no |
| chr17 | 41246533 | 41246596 | Frameshift | c.952_1015delCATAACAGATGGGCTGGAAGTAAGGAAACATGTAATGATAGGCGGACTCCCAGCACAGAAAAAA | His318fs | no | yes |
| chr17 | 41246785 | 41246785 | Stop-gain | c.763G>T | Glu255* | no | yes |
| chr17 | 41246872 | 41246872 | Frameshift | c.676delT | Cys226fs | yes | no |
| chr17 | 41251834 | 41251834 | Stop-gain | c.505C>T | Gln169* | yes | no |
| chr17 | 41256985 | 41256985 | Splice region | c.213-12A>G |  | yes | no |
| chr17 | 41258504 | 41258504 | Missense | c.181T>G | Cys61Gly | yes | yes |
| chr17 | 41258525 | 41258525 | Stop-gain | c.160C>T | Gln54* | yes | no |
| chr17 | 41267764 | 41267765 | Frameshift | c.112_113delAA | Lys38fs | no | yes |
| chr17 | 41267797 | 41267797 | Splice-acceptor | c.81-1G>A |  | no | yes |
| chr17 | 41276032 | 41276032 | Splice-donor | c.80+2T>A |  | no | yes |
| chr17 | 41276045 | 41276046 | Frameshift | c.68_69delAG | Glu23fs | yes | yes |
| chr17 | 41276047 | 41276047 | Frameshift | c.66dupA | Glu23fs | no | yes |
| chr17 | 41276080 | 41276080 | Stop-gained | c.34C>T | Gln12* | yes | no |
