# Supplementary figures and images for: Investigating the effects of additional truncating variants in DNA-repair genes on breast cancer risk in BRCA1-positive women
Source: BMC Cancer. 2019 Aug 8;19:787. doi: 10.1186/s12885-019-5946-0 (PMC6686546; doi:10.1186/s12885-019-5946-0)

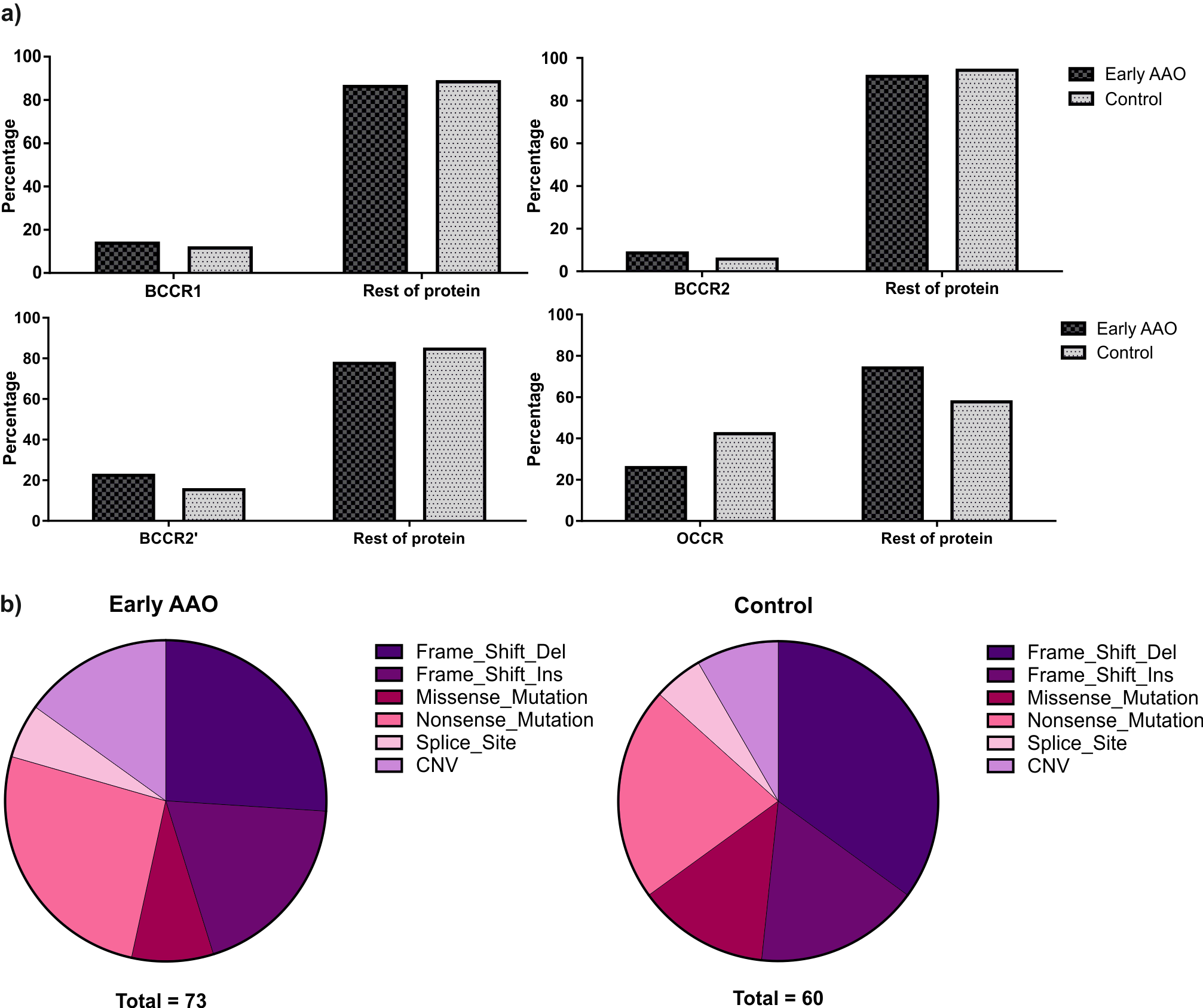

Supplement: Supplementary file 4 — : Figure S1 Comparison of type and location of BRCA1 pathogenic variants in two cohorts: a) Accumulation of pathogenic variants in BCCR (Breast Cancer Cluster Region) and OCCR (Ovarian Cancer Cluster Region) are compared in both cohorts. b) Comparison of type of pathogenic variants in two cohorts; Del: deletion; Ins: insertion; CNV: Copy Number Variation. (TIFF 13270 kb) [file 12885_2019_5946_MOESM4_ESM.tiff]

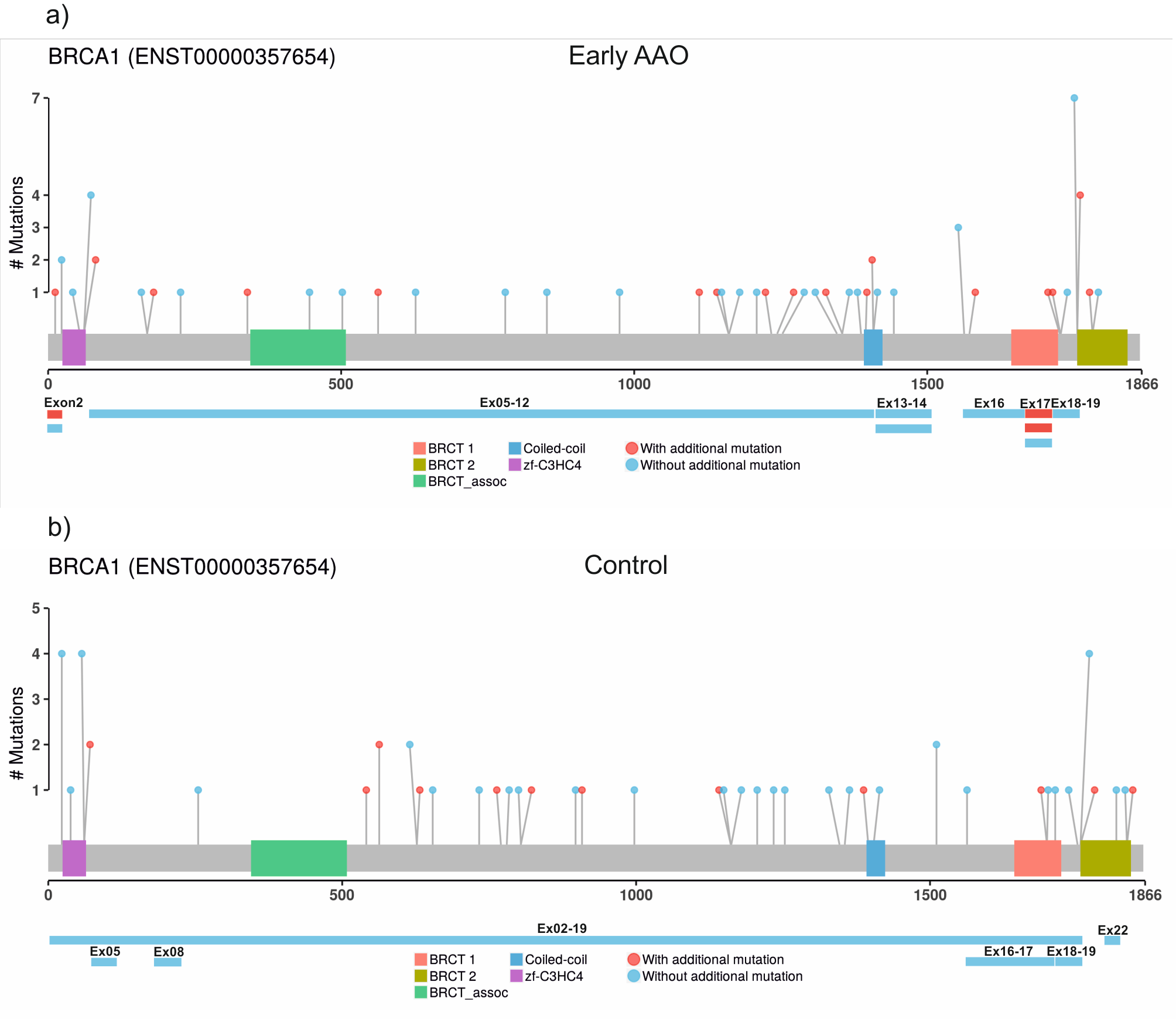

Supplement: Supplementary file 6 — : Figure S2 Additional truncating variants carriers vs non-carriers . The lollipop plot shows the position of BRCA1 pathogenic variants in two cohorts: (a) early AAO and (b) Control cohort; with and without additional truncating variant in DNA-repair genes. X axis shows the functional domain of BRCA1 protein and amino acid position and Y axis demonstrates the number of carriers. Each lollipop represents the location of a BRCA1 pathogenic variant of those with (red) and without (blue) additional truncating variants . Horizontal bars depict the copy number variations of those with (red) and without (blue) additional truncating variant. Splice-site variants are not shown. (TIFF 13653 kb) [file 12885_2019_5946_MOESM6_ESM.tiff]

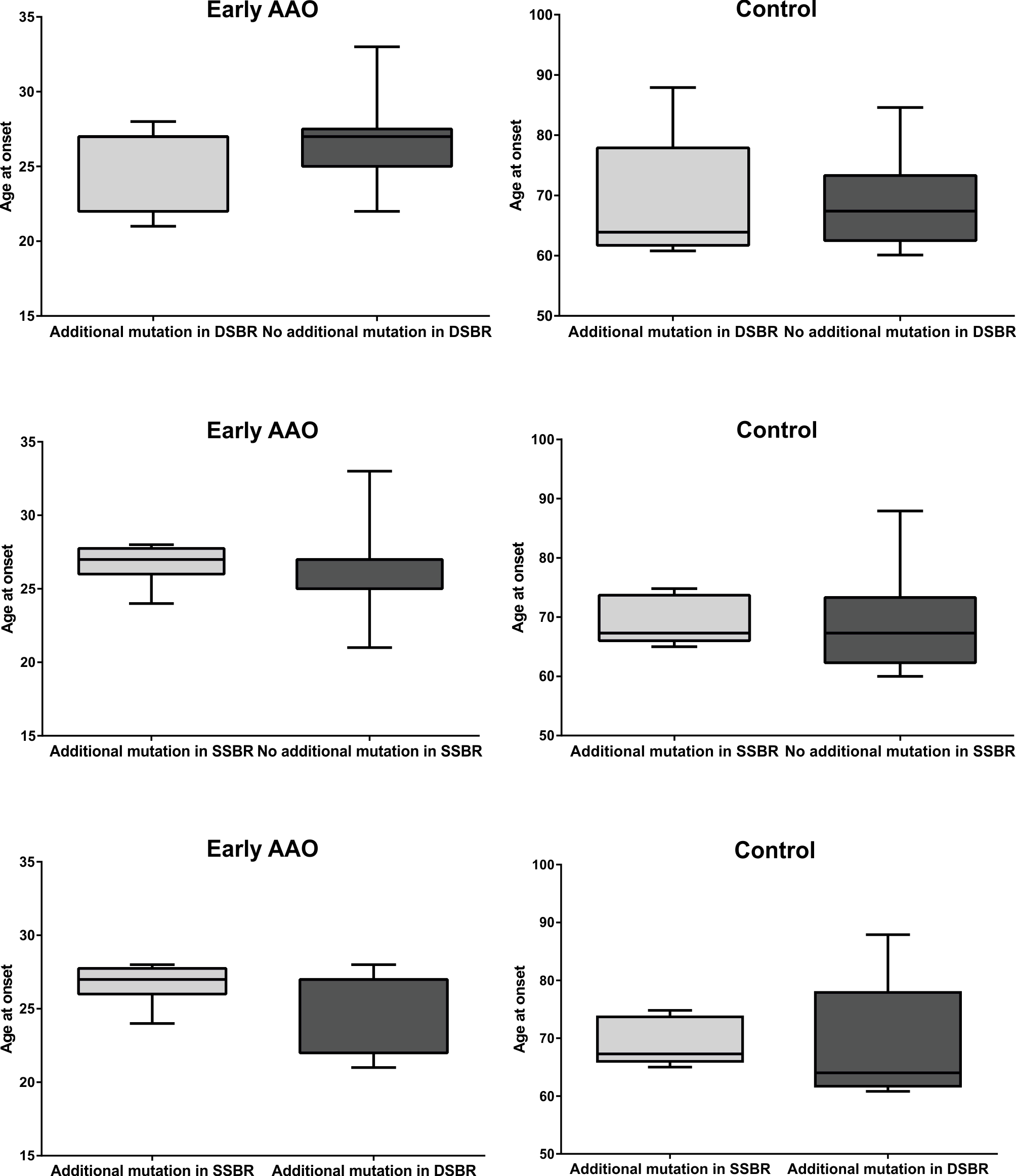

Supplement: Supplementary file 7 — : Figure S3 Comparison of AAO between DSBR/SSBR gene mutation carriers and non-carriers. (TIFF 18234 kb) [file 12885_2019_5946_MOESM7_ESM.tiff]
