# Supplementary material for: Investigating the effects of additional truncating variants in DNA-repair genes on breast cancer risk in BRCA1-positive women
Source: BMC Cancer. 2019 Aug 8;19:787. doi: 10.1186/s12885-019-5946-0 (PMC6686546; doi:10.1186/s12885-019-5946-0)
Supplement: Supplementary file 8 — : Table S5 Comparison of AAO between carriers of DSBR and SSBR truncating variants in both cohorts. DSBR: Double Strand Break Repair; SSBR: Single Strand Break Repair. (DOCX 14 kb) [file 12885_2019_5946_MOESM8_ESM.docx]

**Table S5.** **Comparison of age at onset between carriers of DSBR and SSBR truncating variants in both cohorts.**

|  | Early AAO cohort  Mean age at onset ± SD (95 %-CI) | Control cohort  Mean age at last follow-up ± SD  (95 %-CI) | |
| --- | --- | --- | --- |
| With additional truncating variant | 26.2 ±2.2 (25.3-27.1) | | 68.3±7.4 (64.3-72.3) |
| No additional truncating variant | 26.3±2.1 (25.7-26.9) | | 69.0±7.3 (66.8-71.2) |
| DSBR | | | |
| With additional truncating variant | 25.4± 2.8 (23.0-27.7) | 68.6± 11.1 (54.9-82.4) | |
| No additional truncating variant | 26.6± 1.9 (25.7-27.5) | 67.8± 6.1 (63.7-71.9) | |
| SSBR | | | |
| With additional truncating variant | 26.6± 1.3 (25.5-27.7) | 69.3± 4.2 (62.4-74.5) | |
| No additional truncating variant | 26.1± 2.5 (24.8-27.3) | 67.8± 8.7 (62.0-73.7) | |

DSBR: Double Strand Break Repair; SSBR: Single Strand Break Repair.
