# Supplementary material for: Investigating the effects of additional truncating variants in DNA-repair genes on breast cancer risk in BRCA1-positive women
Source: BMC Cancer. 2019 Aug 8;19:787. doi: 10.1186/s12885-019-5946-0 (PMC6686546; doi:10.1186/s12885-019-5946-0)
Supplement: Supplementary file 9 — : Table S6 Comparison of histopathological characteristics of DNA-repair mutation carriers with non-carriers. There was no significant difference in tumors of patients carrying additional truncating variant in DNA-repair genes compare to non-carriers in each cohort. ER: Estrogen receptor; PR: Progesterone receptor; HER2: Human Epidermal growth factor receptor 2. (DOCX 16 kb) [file 12885_2019_5946_MOESM9_ESM.docx]

**Table S6.** **Comparison of histopathological characteristics of DNA-repair mutation carriers with non- carriers**.

|  | Early age at onset( <35)  n =.73 | | Controls (>60) with cancer manifestation  n = 25 | |
| --- | --- | --- | --- | --- |
|  | **With additional truncating variant**  **n= 26** | **Without additional truncating variant**  **n= 47** | **With additional truncating variant**  **and breast cancer**  **n = 6** | **Without additional truncating variant**  **and breast cancer**  **n = 19** |
| Data Available | 21 | 43 | 5 | 17 |
| ER negativity | 15 (71.4 %)  95 %-CI (47.8-88.7 %) | 32 (74.4 %)  95 %-CI (58.8-86.5 %) | 4 (80.0 %)  95 %-CI (28.4-99.5 %) | 9 (52.9 %)  95 %-CI (27.8-77.0 %) |
| Data Available | 21 | 43 | 5 | 17 |
| PR negativity | 17 (81.0 %)  95 %-CI (58.1-94.6 %) | 35 (81.4 %)  95 %-CI (66.6-91.6 %) | 5 (100 %) | 12 (70.6 %)  95 %-CI (44.0-89.7 %) |
| Data Available | 19 | 33 | 4 | 15 |
| HER2 negativity | 17 (89.5 %)  95 %-CI (66.9-98.7 %) | 32 (97.0 %)  95 %-CI (84.2-99.9 %) | 4 (100 %) | 13 (86.7 %)  95 %-CI (59.5-98.3 %) |
| Data available | 22 | 44 | 5 | 16 |
| Grade 3 | 17 (77.3 %)  95 %-CI (54.6-92.2 %) | 36 (81.8 %)  95 %-CI (67.3-91.8 %) | 3 (60.0 %)  95 %-CI (14.7-94.7 %) | 11 (68.8 %)  95 %-CI (41.3-89.0%) |
| Data Available | 22 | 42 | 5 | 19 |
| Ductal carcinoma | 18 (81.8 %)  95 %-CI (59.7-94.8 %) | 35 (83.3 %)  95 %-CI (68.6-93.0%) | 5 (100 %) | 17 (89.5 %)  95 %-CI (66.9-98.7 %) |

There was no significant difference in tumors of patients carrying additional truncating variant in DNA-repair genes compare to non-carriers in each cohort. ER: Estrogen receptor; PR: Progesterone receptor; HER2: Human Epidermal growth factor receptor 2
