# Supplementary material for: Investigating the effects of additional truncating variants in DNA-repair genes on breast cancer risk in BRCA1-positive women
Source: BMC Cancer. 2019 Aug 8;19:787. doi: 10.1186/s12885-019-5946-0 (PMC6686546; doi:10.1186/s12885-019-5946-0)
Supplement: Supplementary file 10 — : Table S7 The top 8 genes that stood out in the Burden test. q value after FDR correction. (DOCX 13 kb) [file 12885_2019_5946_MOESM10_ESM.docx]

**Table S7**. **The top 8 genes that stood out in the Burden test**.

| Gene | no. affected individual in early AAO cohort | no. affected individual in control cohort | Total no. Early AAO | Total no. Control | p value | q value | Potential effect |
| --- | --- | --- | --- | --- | --- | --- | --- |
| MRE11 | **7** | **0** | **73** | **60** | **0.0093** | **0.9** | **Risk** |
| PTCH1 | **1** | **8** | **73** | **60** | **0.0129** | **0.9** | **Protective** |
| MYBBP1A | **13** | **3** | **73** | **60** | **0.0169** | **0.9** | **Risk** |
| WRN | **7** | **1** | **73** | **60** | **0.0342** | **0.9** | **Risk** |
| TDG | **5** | **0** | **73** | **60** | **0.0409** | **0.9** | **Risk** |
| TP53BP1 | **10** | **3** | **73** | **60** | **0.0415** | **0.9** | **Risk** |
| REV1 | **8** | **2** | **73** | **60** | **0.0488** | **0.9** | **Risk** |

q value after FDR correction
